# Toward Universal Health Coverage: What Socioeconomic and Clinical Factors Influence Health Insurance Coverage and Restrictions in Access to Viral Hepatitis Services in Nasarawa State, Nigeria?

**DOI:** 10.3390/ijerph21101373

**Published:** 2024-10-17

**Authors:** Victor Abiola Adepoju, Donald C. Udah, Chinonye Alioha Ezenwa, Jamiu Ganiyu, Sumaiya Muhammad Lawal, James Ambo Haruna, Qorinah Estiningtyas Sakilah Adnani, Adamu Alhassan Ibrahim

**Affiliations:** 1 Department of HIV and Infectious Diseases, Jhpiego Nigeria, Affiliate of Johns Hopkins University, Abuja 900911, Nigeria; 2 JSI Research & Training Institute Inc. (JSI), Abuja 900911, Nigeria; donald.udah@gmail.com; 3 Department of Strategic Information, Jhpiego Nigeria, Affiliate of Johns Hopkins University, Abuja 900911, Nigeria; tonyalioha@gmail.com; 4 National AIDS/STI and Viral Hepatitis Control Program, Federal Ministry of Health, Abuja 900911, Nigeria; jamiyug@gmail.com; 5 General Hospital Akwanga, Akwanga 960101, Nasarawa State, Nigeria; sumaiyalawal2016@gmail.com; 6 Our Ladies of Apostles Hospital, Akwanga 960101, Nasarawa State, Nigeria; ambojames.jh@gmail.com; 7 Department of Public Health, Faculty of Medicine, Universitas Padjadjaran, Bandung 40161, West Java, Indonesia; qorinah.adnani@unpad.ac.id; 8 Director of Public Health Services, Nasarawa State Ministry of Health, Lafia 950101, Nasarawa State, Nigeria; ibrahima57@yahoo.com

**Keywords:** health insurance, Nigeria, universal health coverage, sociodemographic predictors, viral hepatitis B and C

## Abstract

**Background:** Viral hepatitis B and C (HBV and HCV) pose significant public health concern in Nigeria, where access to healthcare and treatment affordability are limited. This study investigated sociodemographic and clinical predictors of health insurance coverage and access to care among patients with HBV and HCV in Nasarawa State, Nigeria. **Methods:** A cross-sectional facility-based study was conducted at two secondary hospitals in Nasarawa State, Nigeria. Participants included patients diagnosed with HBV, HCV, or both who were ≥18 years old. Data were collected using a structured questionnaire covering sociodemographic and clinical information, health insurance details, and economic impact. Binary logistic regression was used to analyze the relationship between sociodemographic/clinical factors and health insurance status. **Results:** Out of 303 participants, 68% had health insurance, which mostly covered hepatitis screening and vaccination. Significant predictors of health insurance coverage included being aged 36–40 years (adjusted odds ratio [aOR]: 11.01, 95% confidence interval [CI]: 2.38–50.89, *p* = 0.002), having post-secondary education (aOR: 25.2, 95% CI: 9.67–65.68, *p* < 0.001), being employed (aOR: 27.83, 95% CI: 8.85–87.58, *p* < 0.001), and being HIV-positive (aOR: 4.06, 95% CI: 1.55–10.61, *p* = 0.004). Nearly all those insured (99%) faced restrictions in insurance coverage for viral hepatitis services. **Conclusions:** This study reveals that while health insurance coverage is relatively high among viral hepatitis patients in Nasarawa State, significant restrictions hinder access to comprehensive services, especially for vulnerable groups like younger adults, the unemployed, and PLHIV. Key factors influencing coverage include age, education, employment, and HIV status. Expanding benefit packages to include viral hepatitis diagnosis and treatment, raising awareness about viral hepatitis as part of insurance strategy, improving access for underserved populations, and integrating hepatitis services into existing HIV programs with strong policy implementation monitoring frameworks are crucial to advancing universal health coverage and meeting the WHO’s 2030 elimination goals.

## 1. Introduction

Globally, the challenge posed by viral hepatitis B and C (HBV and HCV) is significant and deeply concerning, particularly with the risk of liver cirrhosis and liver cancer. The World Health Organization (WHO) estimated that in 2019, a staggering figure of nearly 296 million people were living with chronic hepatitis B, and an additional 58 million were suffering from chronic hepatitis C globally [1]. Despite the availability of effective treatments and vaccines, the widespread impact of viral hepatitis on mortality and morbidity continues unabated. This is largely attributed to barriers such as the prohibitive cost of treatment and the lack of comprehensive health insurance coverage. Although many people are suffering and dying from viral hepatitis, in 2019, only 10% and 21% are aware of their hepatitis B and C status, respectively. As of 2019, the WHO projects that merely 22% and 62% of people who knew they were infected with HBV and HCV respectively had received treatment.

Reflecting on the situation in the United States, a study revealed a worrying trend, that individuals without health insurance who have HCV are significantly less likely to be aware of their infection compared to those who are insured [2]. This underscores a critical disparity in access to hepatitis care based on the individual’s insurance status. A similar scenario is observed in mainland China, where despite high levels of health insurance coverage, patients with chronic hepatitis C are burdened with substantial out-of-pocket expenses [3]. These findings are a stark reminder of the pressing need for comprehensive insurance coverage to bridge the gap in hepatitis care. In the Republic of Korea, another study reported a significant rise in the total cost of hepatitis B treatment over a period of 13 years [4], while in the United States, despite the increase in treatment rates for hepatitis C among insured individuals, disparities in access to care and the increasing economic burden persist [5]. These studies collectively highlight the financial barriers to managing hepatitis and emphasize the indispensable role of health insurance in facilitating access to treatment.

In Nigeria, the burden of viral hepatitis, coupled with its associated financial costs, presents a grim picture. With a population of 220 million, the National AIDS Indicator and Impact Survey reported the prevalence of 1.1% and 8.1% for hepatitis C and B, respectively. However, this could be up to 13% in Nasarawa State [6,7]. The prevalence rates translate to about 20 million Nigerians infected with viral hepatitis B and C. Nasarawa confronts challenges such as the low disease awareness and high disease burden. To combat this, in 2020, the state government committed to a five-year plan to screen 2.5 million and treat 141,000 people for HCV by 2025. Nasarawa State also provides free HCV services to patients dual infected with hepatitis C and HIV within its HCV micro-elimination strategy.

Nigeria is grappling with the dual burden of communicable and noncommunicable diseases, all within the context of limited healthcare resources and the limitations of universal health coverage. Studies like those conducted in two tertiary hospitals in Rivers State, Nigeria, have shown that the financial cost of managing viral hepatitis in Nigeria is overwhelming, often surpassing the financial capabilities of individuals and families, particularly in areas with limited resources [8,9]. This burden is further compounded by Nigeria’s low minimum wage of NGN 30,000 (USD 79), rendering the cost of hepatitis screening, diagnosis, treatment, and follow-up prohibitively expensive for a large segment of the population [8].

A critical component of this challenge is the issue of health insurance coverage in Nigeria. The National Health Insurance Authority (NHIA) has the mandate to provide universal health coverage, yet unable to reach its full potential. Despite recent efforts to expand the benefit package to cover viral hepatitis diagnosis and treatment at secondary and tertiary facilities, its implementation is not evident on the field. The coverage for viral hepatitis is still limited, leaving many Nigerians to shoulder the high costs of healthcare services out of pocket. This situation is especially complex for patients with viral hepatitis B, who require continuous and often costly medical care. A report from the 2018 Nigeria Demographic and Health Survey indicated that only 3% of Nigerians have any form of health insurance [10]. According to another survey conducted by the Lagos Bureau of Statistics, only 11% of household members in Lagos state are covered by any form of health insurance [11]. The Lancet Nigeria Commission highlighted the challenges in Nigeria’s health system, including low health investment and high out-of-pocket expenditures, which account for a staggering 77% of total health spending. This significantly hampers the progress toward achieving universal health coverage [12].

Despite the well-acknowledged importance of health insurance in managing viral hepatitis, there exists a notable gap in the literature concerning the sociodemographic and clinical predictors of health insurance coverage among HBV and HCV patients in Nigeria. Understanding these predictors is vital for developing targeted interventions aimed at improving a health insurance benefit package and, subsequently, access to viral hepatitis services. This study investigated these predictors within the Nigerian context, where the burden of viral hepatitis is high, and the financial cost of care presents a formidable barrier to effective disease management.

The goals of this study are twofold: to identify the sociodemographic and clinical factors that predict health insurance coverage among patients with viral hepatitis B and C in Nigeria, and to understand how these factors influence access to and utilization of healthcare services for viral hepatitis. By addressing these objectives, this study seeks to contribute to the broader goal of achieving universal health coverage and reducing the burden of viral hepatitis in Nigeria. This research is particularly relevant in the context of Nigeria’s ongoing efforts to reform its health system and expand health insurance coverage to include more comprehensive services through the Honorable Minister for Health and Social Welfare’s Nigeria Health Sector Renewal Investment Initiative and the Sector-wide Approach (SWAp).

## 2. Materials and Methods

### 2.1. Study Design

This is a cross-sectional, facility-based study, conducted at the General Hospital Akwanga (GH Akwanga) and Our Lady of Apostle Hospitals (OLA) in Nasarawa State, Nigeria, during November–December 2023.

### 2.2. Study Setting

Nasarawa State is in North Central Nigeria with approximately 4 million inhabitants. Nasarawa state has a high prevalence of viral hepatitis, with 14% for HBV and 13.2% for HCV. The selection of Nasarawa State was strategic, given its high burden of both viral hepatitis B and C. GH Akwanga operates as a government-owned secondary health facility, while OLA is a faith-based institution. Both facilities are known for their comprehensive prevention, care, and treatment services for HIV, as well as viral hepatitis B and C. They both offer diagnostic and testing services for tuberculosis, including GeneXpert machines optimized for viral load testing for viral hepatitis.

### 2.3. National and State Health Insurance Programs in Nigeria

The new National Health Insurance Act (NHIA) 2022, recently replaced the older 1999 Act. The aim is to provide mandatory health insurance for all Nigerians and legal residents. NHIA focuses on integrating and regulating health insurance schemes. It includes a fund for vulnerable groups and promotes the establishment of state health insurance schemes. In 2019, about 19 states were implementing these schemes [13]. These state schemes, funded partly by state revenues, aim to cover the poor and vulnerable. The Nasarawa State Health Insurance Agency (NASHIA) was established under the law in 2019. By September 2023, NASHIA has successfully enrolled over 188,000 residents in its health insurance scheme [14]. The beneficiaries, registered under various packages like public sector, informal sector, vulnerable groups, and students, have been accessing healthcare services across the state. The Nasarawa State Government has implemented a compulsory health insurance policy for all state and local government employees. This mandate includes automatic coverage extension to an employee’s spouse and up to four biological children 18 years old or less, under the same plan. The annual premium for this coverage is established at NGN 15,000 (USD 10). The policy is designed with adaptability and equity in mind, which allows the inclusion of additional dependents beyond the immediate nuclear family, such as older children, elderly parents, or other dependents, with the condition of an additional fee determined by NASHIA. Moreover, the Basic Healthcare Provision Fund mechanism is strategically utilized to subsidize health insurance, with a focus on aiding the more vulnerable segments of the population. This strategy aims to guarantees a comprehensive reach of healthcare benefits, catering to a wide array of demographic groups within Nasarawa State.

### 2.4. Participants

To be included in this study, participants must be aged 18 years and above and diagnosed with hepatitis B, C, or both. These participants were either in the process of receiving, awaiting, or had completed treatment at either GH Akwanga or OLA hospitals. Individuals who tested negative for HbsAg or anti-HCV antibodies were excluded from this study.

### 2.5. Data Collection

Data collection took place during November–December 2023. Two trained data collectors were deployed to administer an electronic, interviewer-assisted questionnaire, initially developed using Google Forms. To enhance data reliability and validity, this questionnaire was pilot tested at a health facility not included in this study. Subsequently, feedbacks and revisions were received, collated, and integrated into the final version before the actual administration. The questionnaire was designed to capture a wide array of sociodemographic and clinical information, alongside details on health insurance and the economic impact of viral hepatitis treatment. Moreover, patient medical records were meticulously reviewed to validate the diagnoses of hepatitis B or C in alignment with the inclusion criteria of this study. We electronically obtained informed consent from all participants in this study through verbal consent administered by the interviewer. The consent form was read aloud from the first page of the questionnaire, and participants were required to provide their consent before proceeding. A mandatory consent checkbox was included at the beginning of the Google Form, with a description of the study’s purpose appearing right before the consent block. Once a participant gave their verbal consent, the interviewer checked the ‘yes’ box before allowing them to continue to the questions on the next page of the survey.

### 2.6. Questionnaire Structure

There was a total of 33 questions in the questionnaire. The questionnaire was divided into four distinct sections: 

Section 1 (14 questions): Gathered sociodemographic and clinical information, including age, sex, area of residence, marital status, employment status, monthly household income, education level, population type, HIV status, and HBV/HCV status. 

Section 2 (5 questions): Collected information on health insurance coverage and specific viral hepatitis services, the type of health insurance and any accompanying restrictions. 

Section 3 (8 questions): Assessed the cost and affordability aspects of viral hepatitis services covered by health insurance. 

Section 4 (6 questions): Gathered information on comorbidity, patient experience, and recommendations for insurance coverage.

The responses from the electronic questionnaire were saved in csv file format and exported into Statistical Package for Social Sciences (SPSS) version 25 for efficient handling and analysis. Categorical variables within the data set were systematically coded to facilitate quantitative analysis in SPSS. Each categorical variable was assigned a numeric code representing different categories or responses. For open-ended responses, a systematic coding process was used to transform the qualitative aspects of the data set, making it ready for analysis using SPSS software. This involved assigning numeric codes to specific themes or categories derived from the qualitative data.

The dependent variable included the health insurance status of the participants, while independent variables included sociodemographic and clinical characteristics. Quantitative variables such as age, monthly household income, and education level were categorized for analysis. Age was grouped into predefined ranges (e.g., 18–30, 31–45, etc.) to facilitate age-specific analysis. Monthly household income was categorized into brackets (e.g., less than USD 500, USD 500–1000, and >USD 1000) to assess the economic impact across different income levels. Education level was classified into categories such as no formal education, primary, secondary, and tertiary education. These groupings were chosen to enable a nuanced understanding of the sociodemographic factors influencing health insurance coverage among the study participants.

### 2.7. Bias and Study Size

This study acknowledged the potential for recall bias and implemented strategies to minimize its impact. The sample size was determined using Cochran’s formula for cross-sectional studies. The analyses also factored in a 95% confidence level, a 50% proportion of catastrophic expenditure, a margin of error of 10%, and a nonresponse rate of 10% [15]. This calculation yielded a minimum sample size of 304 patients with viral hepatitis B and C.

### 2.8. Statistical Analysis

Data were analyzed using the SPSS version 25. Descriptive statistics were presented in tables and charts, and logistic multivariate regression analysis was employed to examine the relationship between sociodemographic/clinical variables and health insurance status. In this study, missing data were addressed using listwise deletion. This approach was chosen because the amount of missing data was minimal. Out of the total responses, nine were from individuals who tested negative or had missing results for both HBV and HCV, thus not meeting the inclusion criteria. These responses were excluded from the final analysis. The impact of missing data was considered negligible on the overall study findings.

### 2.9. Ethics

This study received ethical clearance before its commencement. The Nigerian Health Research Ethics Committee (NHREC) approved the study protocol, with the approval number NHREC/01/01/2007 dated 15 September 2023. Participation in this study was voluntary. Prior to recruitment, each participant gave electronic verbal consent, administered by the interviewer through a questionnaire. The consent form, read aloud from the first page of the questionnaire, included a checkbox marked ‘yes’ by the interviewer upon consent, before proceeding to answer questions on subsequent pages of the Google Form. This study is part of a larger research initiative titled ‘A multidimensional assessment of viral hepatitis in Nasarawa State, Nigeria’.

## 3. Results

### 3.1. Sociodemographic Characteristics of Participants

The majority of the study population were females (62%), with the largest age group (30%) being 36–40 years. Most participants were classified as ever married (84%), and over half were employed (57%). The largest category of the respondents had a monthly household income less than USD 500 (66%) and had attained tertiary education (64%). About 58% of the participants resided in urban areas while 42% resided in rural areas. Comorbid conditions were present in 23% of the participants. About 56% were HIV positive. Hepatitis B was found in 34% of the participants, whereas hepatitis C was prevalent in 80% (Table 1).

### 3.2. Health Insurance Status and Coverage

Approximately two-thirds of the participants (68%) had health insurance while 32% did not have health insurance coverage (Table 2). Among those insured, the majority (87%) were covered by government (state or federal) insurance schemes, and nearly all (99%) faced restrictions in insurance coverage for viral hepatitis services. Most (69%) respondents reported experiencing household poverty due to hepatitis treatment costs. Hepatitis status indicate that HCV mono-infection was the most prevalent (66%), followed by HBV mono-infection (21%) and HBV/HCV co-infection (13%). Among those with HIV, 78% had HIV-HCV co-infection, 14% had HIV-HBV-HCV triple-infection, and 8% had HIV-HBV co-infection. Regarding treatment status, just over half of the participants (55%) were receiving or had completed treatment for hepatitis, while 45% were not on treatment.

**Figure 1:**
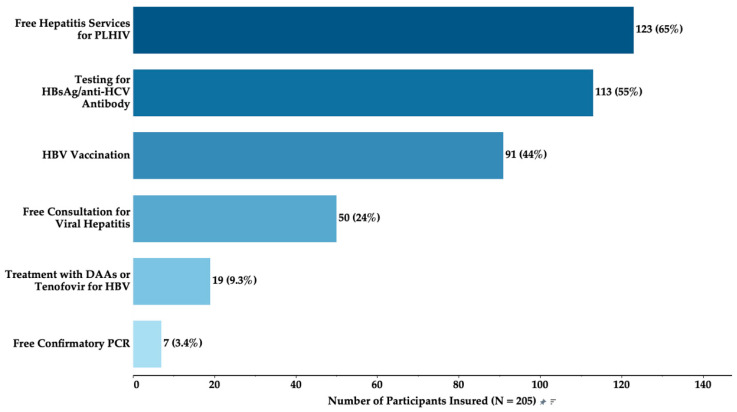
Hepatitis services covered by health insurance among individuals with health insurance.

A variety of hepatitis services were covered by health insurance among participants insured (Figure 1). The most common service covered was testing for the HBsAg/anti-HCV antibody (55%), followed by HBV vaccination (44%). Free hepatitis services for PLHIV were available to 65% of participants. Free consultation for viral hepatitis was available to 24%, while treatment with DAAs for HCV or Tenofovir for HBV was accessible to 9.3%. Only 3.4% had access to free confirmatory PCR tests.

### 3.3. Association between Sociodemographic Factors and Health Insurance Coverage

There was a significant association between health insurance coverage and age group (chi-squared test = 22.0, *p* < 0.001), marital status (chi-squared test = 7.4, *p* = 0.007), employment status (chi-squared test = 126.5, *p* < 0.001), monthly household income (chi-squared test = 24.9, *p* < 0.001), education level (chi-squared test = 119.7, *p* < 0.001), HIV status (chi-squared test = 19.1, *p* < 0.001), hepatitis status (chi-squared test = 20.8, *p* < 0.001), and HIV with HBV and/or HCV co-infection (chi-squared test = 6.0, *p* = 0.043) (Table 3). The proportion of insured participants in the 36–40 years group (34%) was higher than that of the uninsured (20%), whereas the proportion of uninsured participants was higher in the 18–30 years group (22%), compared to the insured (7%). Moreover, the proportion of ever-married individuals was higher among the insured (88%) compared to the uninsured (76%). The employed made up a higher proportion of the insured (80%) compared to the uninsured (11%). There was a higher proportion of insured participants earning more than USD 1000 (12%) compared to the uninsured (2%). Similarly, 85% of insured participants had post-secondary education, compared to 20% of the uninsured. A greater proportion of insured participants (64%) were HIV-positive compared to 38% of the uninsured. Moreover, there was a higher proportion of HCV mono-infection among the insured (75%), compared to the uninsured (49%). Interestingly, a greater proportion of insured participants (82%) had HIV-HCV co-infection compared to 65% of the uninsured. Gender and area of residence did not show a significant association with insurance status.

Some sociodemographic factors clearly influenced health insurance coverage in the binary logistic regression analysis (Table 4). Those aged 36–40 years were significantly more likely than those aged 18–30 years to have insurance (adjusted OR = 11.01, 95% CI = 2.38–50.89, *p* = 0.002); those aged 41–45 years were similarly more likely (adjusted OR = 11.34, 95% CI = 2.09–61.38, *p* = 0.005). Employed individuals were significantly more likely to have insurance than unemployed people; therefore, employment status was a substantial predictor (adjusted OR = 27.83, 95% CI = 8.85–87.58, *p* = 0.001). Another important element was post-secondary education, which showed a considerable positive link with insurance coverage (adjusted OR = 25.2, 95% CI = 9.67–65.68, *p* = 0.001). Participants who tested HIV positive were also more likely to have insurance than those who were HIV-negative (adjusted OR = 4.06, 95% CI = 1.55–10.61, *p* = 0.004). In contrast, in the adjusted model, gender, income, area of residence, and comorbid conditions did not reveal any significant associations.

## 4. Discussion

Our study found that 68% of patients diagnosed with viral hepatitis in Nasarawa State, Nigeria, are covered by health insurance. However, nearly all insured individuals (99%) faced significant restrictions when accessing vital viral hepatitis services. This reflects broader patterns observed in other regions, like the United States, mainland China, the Republic of Korea, and Europe, where vulnerable populations also encounter restricted access to hepatitis B and C treatment [3,4,16,17,18]. Even those with insurance coverage could not access comprehensive viral hepatitis care, which could intensify the financial burden and contribute to delayed or incomplete treatment. The financial burden of hepatitis management remains substantial in Nigeria, where the minimum wage hovers around USD 30–50 per month, far from enough to cover confirmatory test and treatment costs [8]. Individuals with viral hepatitis B and C in Nigeria often struggle with prohibitive out-of-pocket expenses for essential services, including PCR tests and antiviral treatments like DAAs or Tenofovir. In addition, preventive measures like HBV vaccination frequently require self-payment, further worsening inequities in access to these services. Studies from the Hepatitis B Foundation and Furukawa et al. [19,20] reveal similar issues in the United States, where insurance often fails to fully cover hepatitis care, placing a heavy financial strain on patients seeking services. Addressing these barriers through insurance policy reforms and facilitating nationwide access to global price reductions for viral hepatitis commodities including essential medications in Nigeria, is critical to advancing progress toward the WHO’s 2030 viral hepatitis elimination goals.

While our study found a high insurance coverage rate in Nasarawa State, it is crucial to recognize that this might not represent the national average. The overall coverage for health insurance in Nigeria remains low and ranges between 2.1% and 9.5% [21,22] and 0.5% to 57.9% across various African countries [23,24]. This substantial gap in the existing health insurance systems at a national level shows the urgent need for increased investment in health insurance reforms. Expanding coverage to include vulnerable populations, particularly younger adults and the elderly, and addressing the gaps in hepatitis service coverage are essential steps toward achieving universal health coverage and reducing the burden of viral hepatitis in Nigeria.

In Nigeria, health insurance coverage, at national and state levels, reveals gaps in addressing viral hepatitis across specific age groups. The NHIA Operational Guidelines 2023 reveal disparities in health insurance coverage across different age groups, which may particularly exclude the demographics that are typically at risk of viral hepatitis [25]. Per the operational guidelines, the plan covers employees (enrollees), their spouses, and up to four biological children under the age of 18 years. This framework, however, overlooks dependents over 18 years, unless they are included in specialized schemes like the Tertiary Institution Social Health Insurance Program (TISHIP) or any other available mechanisms after paying an additional fee. Our study also identifies key sociodemographic and clinical predictors of health insurance coverage. Individuals aged 36–40 and 41–45 were significantly more likely to have insurance compared to younger adults (18–30 years) and the elderly (>45 years). Younger adults might face barriers to accessing health insurance due to factors like unemployment or lack of stable income, while the elderly might be excluded due to age-related restrictions or higher premiums. The strong association between post-secondary education and employment with insurance coverage in our study further amplifies the role of socioeconomic factors in healthcare access. As observed in other studies from Nigeria, Ghana, Nepal, China, and the United States, higher education and employment status often correlate with better access to health insurance [2,3,20,21,22,23,24,26].

Our findings also reveal a significant association between HIV-positive status and health insurance coverage in Nasarawa State. This observation is likely due to the state’s proactive HCV micro-elimination strategy, 2020–2024 [27], which prioritizes free HCV services for individuals co-infected with HIV and hepatitis C. Also, the two hospitals included in this study have been accredited to provide not only comprehensive viral hepatitis prevention, care, and treatment, but also health insurance services explaining the high enrolment rates in these settings coupled with the mandatory nature of health insurance in Nasarawa State.

The Lancet Nigeria Commission’s call for a holistic, whole-of-government approach to universal health coverage and health insurance systems in Nigeria, supported by contributions and taxation, to address health inequities and economic challenges [12] strongly resonates with these findings. The sector-wide approach (SWAp) is the initiative by the Government of Nigeria which aims to unlock the value chain and promote local manufacturing of health commodities. If implemented, this will be a promising long-term strategy for improving healthcare access and affordability. However, in the immediate and medium term, critical reforms are needed to ensure that the benefits of health insurance reach those most in need without restrictions. Expanding health insurance benefit packages to include comprehensive viral hepatitis diagnosis and treatment, particularly for vulnerable populations like younger adults, the elderly, the unemployed, and PLHIV, is crucial. Removing restrictions on access to hepatitis treatment for PLHIV and other vulnerable groups and integrating hepatitis services into existing HIV programs are essential steps toward equitable and effective healthcare delivery. In addition, improving access to HBV vaccination and making the vaccine available and accessible free-of-charge for infants and adults, especially those at high risk, could significantly reduce the burden of HBV-related liver cancer. Furthermore, our findings on insurance patterns among hospital care seekers provide important insights for targeted policy interventions, particularly those directed at raising awareness among individuals unaware of their viral hepatitis status. Understanding that raising awareness about disease status could increase insurance uptake can provide policymakers with actionable recommendations to improve health coverage in affected populations, thereby enhancing the relevance of our sample to broader health policy discussions.

This study has some limitations, which include the potential for recall bias, the inherent constraints of a cross-sectional survey design, and the focus on two hospitals, both of which are equipped with insurance accreditation. Notwithstanding, the findings are relevant and generalizable to the study setting. Learning from successful nationwide elimination campaigns in countries like Egypt and Rwanda, Nigeria can leverage its SWAp strategy to achieve its goal of eliminating viral hepatitis. Investing in diagnostics and treatment, coupled with comprehensive health insurance reforms and implementation monitoring frameworks, will be key to unlocking the value chain and ensuring that all Nigerians have access to the viral hepatitis prevention, care, and treatment services that they need. We recommend that future studies expand the study coverage to other states in Nigeria, which could result in a larger sample size, potentially improving the precision of estimates, particularly for variables such as age, education level, and employment status, where subgroup variability may have contributed to wider confidence intervals.

In conclusion, this study highlights the complex interplay of factors influencing health insurance coverage and access to hepatitis care in Nigeria. While progress has been made in Nasarawa State, significant challenges remain. By adopting a multifaceted approach that addresses both immediate needs and long-term goals, Nigeria can move closer to achieving universal health coverage and eliminating viral hepatitis as a public health threat.

## Figures and Tables

**Table 1:** Sociodemographic and clinical characteristics.

Characteristic	n (%) ^1^
**Gender**	
Female	189 (62%)
Male	114 (38%)
**Age Group**	
36–40 yrs	90 (30%)
>45 yrs	66 (22%)
41–45 yrs	62 (20%)
31–35 yrs	49 (16%)
18–30 yrs	36 (12%)
**Marital Status**	
Ever Married	254 (84%)
Single	49 (16%)
**Employment Status**	
Employed	174 (57%)
Unemployed	129 (43%)
**Monthly Household Income (USD)**	
<500	201 (66%)
500–1000	76 (25%)
>1000	26 (9%)
**Education Level**	
Post-secondary education	194 (64%)
No post-secondary education	109 (36%)
**Area of Residence**	
Urban	177 (58%)
Rural	126 (42%)
**Comorbid Conditions**	71 (23%)
**HIV Status**	
Positive	169 (56%)
Negative	134 (44%)
**HBV+ Status**	102 (34%)
**HCV+ Status**	241 (80%)

^1^ number of participants (n) and the corresponding percentage (%) within the specified group.

**Table 2:** Health insurance coverage and hepatitis status.

Characteristic	Frequency ^1^
**Health Insurance Status**	205 (68%)
**Insurance Restriction for Hepatitis**	
Yes	202 (99%)
No	3 (1%)
**Type of Health Insurance**	
Government (State)	129 (63%)
Government (Federal)	49 (24%)
Community Based Health Insurance	15 (7%)
Private	6 (3%)
Employer-based	5 (2%)
Others	1 (1%)
**Household Poverty due to Hepatitis Treatment**	196 (69%)
**Hepatitis Status**	
HCV mono-infection	201 (66%)
HBV mono-infection	62 (21%)
HBV/HCV co-infection	40 (13%)
**HBV and/or HCV in HIV**	
HIV-HCV co-infection	132 (78%)
HIV-HBV-HCV co-infection	24 (14%)
HIV-HBV co-infection	13 (8%)
**HBV/HCV Treatment Status**	
Receiving or completed treatment	168 (55%)
Not on treatment	135 (45%)

^1^ number of participants (n) and the corresponding percentage (%) within the specified group.

**Table 3:** Associations between health insurance coverage and sociodemographic and clinical characteristics.

Characteristic	Health Insurance Coverage		*p*-Value ^2^
	Yes, n = 205 ^1^	No, n = 98 ^1^	
**Gender**			0.14
Female	122 (60%)	67 (68%)	
Male	83 (40%)	31 (32%)	
**Age Group**			<0.001
36–40 years	70 (34%)	20 (20%)	
>45 years	39 (19%)	27 (28%)	
41–45 years	46 (22%)	16 (16%)	
31–35 years	36 (18%)	13 (13%)	
18–30 years	14 (7%)	22 (22%)	
**Marital Status**			0.007
Ever Married	180 (88%)	74 (76%)	
Single	25 (12%)	24 (24%)	
**Employment Status**			<0.001
Employed	163 (80%)	11 (11%)	
Unemployed	42 (20%)	87 (89%)	
**Monthly Household Income (USD)**			<0.001
<500	117 (57%)	84 (86%)	
500–1000	64 (31%)	12 (12%)	
>1000	24 (12%)	2 (2.0%)	
**Education Level**			<0.001
Post-secondary education	174 (85%)	20 (20%)	
No post-secondary education	31 (15%)	78 (80%)	
**Area of Residence**			0.3
Urban	116 (57%)	61 (62%)	
Rural	89 (43%)	37 (38%)	
**HIV Status**			<0.001
Positive	132 (64%)	37 (38%)	
Negative	73 (36%)	61 (62%)	
**Hepatitis Status**			<0.001
HCV mono-infection	153 (75%)	48 (49%)	
HBV mono-infection	29 (14%)	33 (34%)	
HBV/HCV co-infection	23 (11%)	17 (17%)	
**HIV with HBV and/or HCV co-infection**			0.043
HIV-HCV co-infection	108 (82%)	24 (65%)	
HIV-HBV-HCV co-infection	17 (13%)	7 (19%)	
HIV-HBV co-infection	7 (5%)	6 (16%)	
**HBV/HCV Treatment Status**			0.4
Receiving or completed treatment	110 (54%)	58 (59%)	
Not on treatment	95 (46%)	40 (41%)	

^1^ number of participants (n) and the corresponding percentage (%) within the specified group; ^2^ Pearson’s chi-squared test; Fisher’s exact test.

**Table 4:** Binary logistic regression analysis assessing the influence of sociodemographic factors on health insurance coverage.

Variable	Crude OR (95% CI)	*p*-Value	Adjusted OR (95% CI)	*p*-Value
Gender				
Male	Ref.		Ref.	
Female	0.68 (0.41–1.13)	0.14	1.32 (0.54–3.23)	0.54
Age Group				
18–30 years	Ref.		Ref.	
31–35 years	4.35 (1.73–10.95)	0.002	2.61 (0.59–11.56)	0.21
36–40 years	5.5 (2.39–12.67)	<0.001	11.01 (2.38–50.88)	0.002
41–45 years	4.52 (1.88–10.88)	0.001	11.34 (2.09–61.38)	0.005
>45 years	2.27 (0.99–5.21)	0.05	4.8 (0.95–24.13)	0.06
Marital Status				
Single	Ref.		Ref.	
Ever Married	2.34 (1.25–4.35)	0.01	0.69 (0.22–2.16)	0.52
Employment Status				
Unemployed	Ref.		Ref.	
Employed	30.69 (15.05–62.62)	0.001	27.83 (8.85–87.58)	<0.001
Monthly Household Income (USD)				
<500	Ref.		Ref.	
500–1000	3.83 (1.95–7.54)	<0.001	0.66 (0.19–2.33)	0.52
>1000	8.62 (1.98–37.45)	0.004	0.38 (0.05–2.6)	0.32
Education Level				
No post-secondary education	Ref.		Ref.	
Post-secondary education	21.89 (11.75–40.79)	<0.001	25.2 (9.67–65.68)	<0.001
Area of Residence				
Urban	Ref.		Ref.	
Rural	0.79 (0.48–1.29)	0.35	0.6 (0.23–1.6)	0.31
HIV Status				
Negative	Ref.		Ref.	
Positive	2.98 (1.81–4.91)	<0.001	4.06 (1.55–10.61)	0.004
HBV/HCV Treatment Status				
Not on treatment	Ref.		Ref.	
Receiving or completed treatment	0.8 (0.49–1.3)	0.37	2.35 (0.96–5.75)	0.06
Comorbid Conditions				
No	Ref.		Ref.	
Yes	1.18 (0.66–2.11)	0.57	0.55 (0.19–1.55)	0.26

CI: confidence interval, OR: odds ratio.

## Data Availability

The data presented in this study are available on request from the corresponding author. The data are not publicly available due to privacy restrictions.
